# Prospects and Dilemmas of Endovascular Treatment for Vertebrobasilar Dolichoectasia

**DOI:** 10.3389/fneur.2022.895527

**Published:** 2022-07-05

**Authors:** Yiheng Wang, Jinlu Yu

**Affiliations:** Department of Neurosurgery, First Hospital of Jilin University, Changchun, China

**Keywords:** vertebrobasilar dolichoectasia, endovascular treatment, aneurysm, prognosis, complication

## Abstract

Vertebrobasilar dolichoectasia (VBD) is characterized by significant expansion, elongation, and tortuosity of the basilar artery and vertebral artery. Certain highly selected cases of VBD can require intervention. Recent advances in endovascular treatment (EVT) have renewed hope for patients with VBD. However, which cases of VBD can benefit from EVT still needs to be determined. Currently, little is known regarding this matter. Therefore, we performed a review of the literature from a PubMed search and cataloged our experience regarding the classification and natural history of VBD and the prospects, prognosis and complications of EVT. The findings can be summarized as follows: for asymptomatic VBD, “wait and see” or medical management may be a reasonable strategy. EVT may only be effective in certain patients. For saccular aneurysms in VBD, especially ruptured aneurysms, EVT is reasonable. For fusiform aneurysms in VBD, EVT can carry high complication rates and should be recommended with caution. For stenting reconstruction in VBD, the effect is uncertain. For the future of EVT of VBD, randomized controlled trials and the development of neurointerventional products are worth pursuing, but EVT in VBD still has a long way to go.

## Introduction

Vertebrobasilar dolichoectasia (VBD) is uncommon, with a prevalence ranging from 7.6 to 18.8% in patients with stroke and 1.3–4.4% in the general population (1, 2). It is characterized by significant expansion, elongation, and tortuosity of the basilar artery (BA) and vertebral artery (VA) (3, 4). The exact etiology of VBD is unknown; however, it is thought to be associated with atherosclerosis, hypertension, collagen vascular disease, polycystic kidney disease, Marfan syndrome, Fabry disease, neurofibromatosis type I, etc. (5, 6). The primary mechanism of VBD is now believed to be aberrant vascular remodeling and abnormal connective tissue within the arterial wall due to an imbalance between matrix metalloproteinases and antiprotease activity in the connective tissue (7–10). Pathologically, VBD may present with a mixed cycle of clotting and hemorrhage within the vessel wall (11, 12).

**Figure 1:**
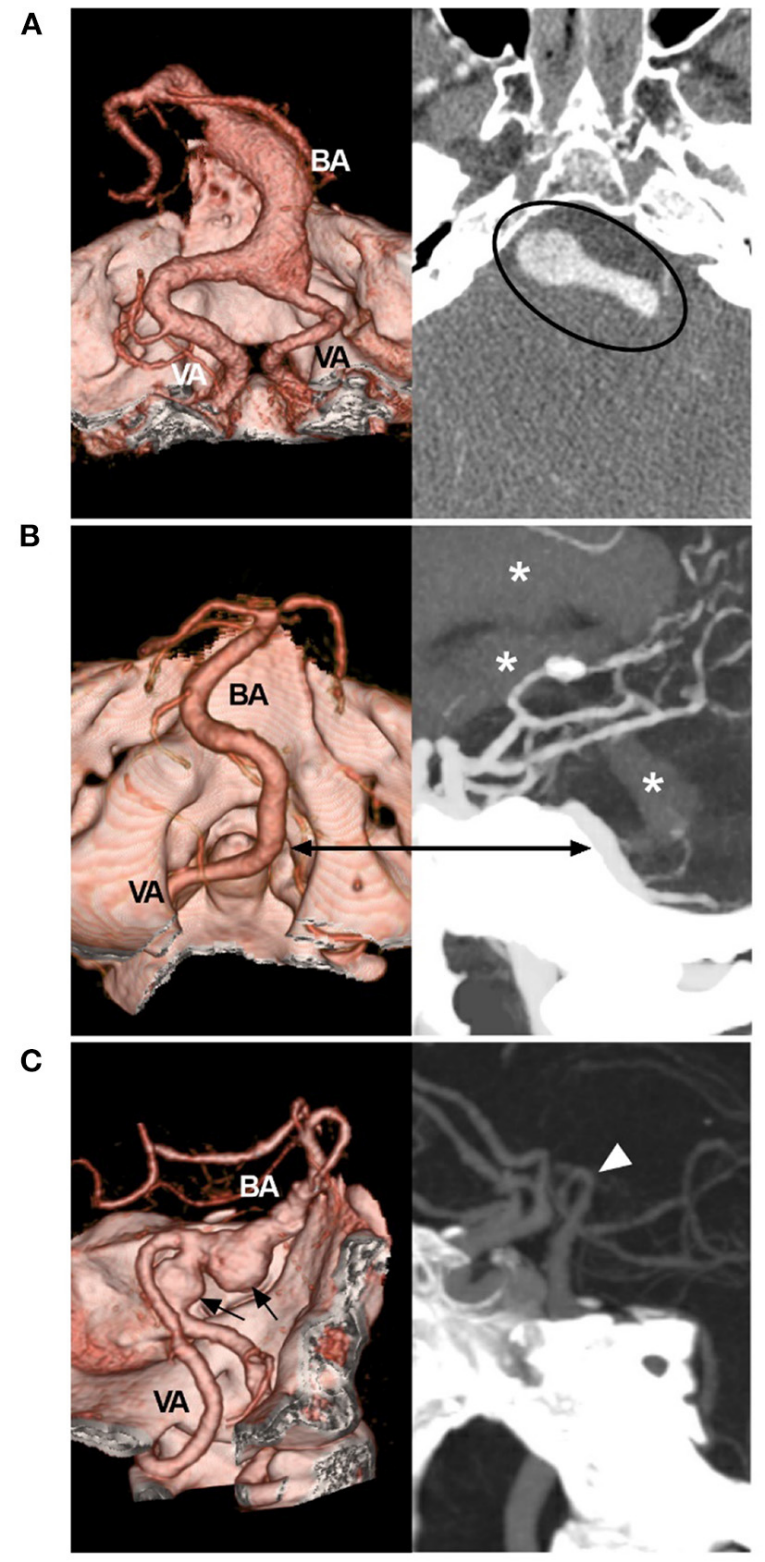
Symptomatic VBD on CTA. **(A)** Left: CTA reconstruction image showing a dilated VBD with mass effect; right: MIP image showing the VBD compressing the brainstem (circle). **(B)** CTA reconstruction image (left) and MIP image (right) of the rupture point of the VBD (bidirectional arrow); asterisks indicate hemorrhage extension from the fourth ventricle to the third and lateral ventricles. **(C)** Left: CTA reconstruction image showing VBD with two aneurysms (arrows); right: MIP image showing the BA bifurcation (arrowhead) reaching the floor of the third ventricle. BA, basilar artery; CTA, computed tomography angiography; MIP, maximum intensity projection; VA, vertebral artery; VBD, vertebrobasilar dolichoectasia.

Most cases of VBD are asymptomatic and diagnosed incidentally (13). When VBD causes elongation and dilation, vertebrobasilar arteries will be distorted, and the blood flow velocity will be reduced and become symptomatic, presenting either with vascular symptoms such as episodes of transient ischemic attacks, ischemic strokes, subarachnoid hemorrhages or with compressive symptoms related to brainstem or cranial nerve compression (Figures 1A,B) (1, 5, 14–17). Of the above symptoms, brainstem and cerebellar ischemia are common, and rupture is uncommon (15, 18–20). In some cases, VBD can result in aneurysm formation (Figure 1C) (21).

Some types of VBD may require treatment, including microsurgery and endovascular treatment (EVT) (22, 23). Recent advances in EVT, especially flow diverting stents (FDSs), have renewed hope for an effective solution to VBD. However, currently, little is known regarding the effect of EVT on VBD. EVT is recommended for this disease without clear, quantifiable criteria. Therefore, we felt it necessary to conduct a literature review from a PubMed search and to recount our experience with the treatment. In this review, some important images and educational cases have been provided.

## Imaging Definition and Classification

### Diagnostic Tools

Several imaging modalities were used to diagnose VBD, including digital subtraction angiography (DSA), computed tomography (CT) angiography (CTA), and magnetic resonance (MR) angiography (MRA). Of them, DSA is still considered to be the gold standard, while MRA is cost-effective and widely available for the diagnosis of VBD (6, 24). CTA can reveal the relationship between the vertebrobasilar trunk and bone structures in VBD (4, 25). On CT and MR images, intraluminal thrombus or calcification and brain parenchymal changes can be well-assessed (5). Transcranial Doppler is useful to study blood flow in VBD (26).

### VBD Definition

To date, there has been no consistent imaging standard to define and classify VBD (27). In 2004, using MRA, Ubogu and Zaidat defined VBD as a BA length >29.5 mm with a >10 mm lateral shift from the vertical line between the initial point and BA tip and an intracranial VA length >23.5 mm with a >10 mm shift of either VA from the line between the initial point of the intracranial VA and the initial point of the BA (28). In 2005, according to vertebrobasilar artery morphology on arteriography and cross-sectional imaging, Flemming et al. defined VBD as uniform dilatation of BA >1.5 times normal involving either the entire BA or VA or both, with any degree of tortuosity (29). When VBD can be associated with superimposed aneurysms, it is called the transitional type (29).

Ubogu and Zaidat (28) and Flemming et al. (29) did not classify or grade cases of VBD. An exact grading system to judge VBD severity is necessary. Using high-resolution CT, Smoker et al. proposed a scoring system for BA as follows: the height of BA bifurcation was scored as 1 (within the suprasellar cistern), 2 (at the level of the third ventricle floor) or 3 (indenting and elevating the third ventricle floor), and lateral displacement of the BA was scored as 1 (medial to lateral margin of clivus or dorsum sellae), 2 (lateral to lateral margin of clivus or dorsum sellae), or 3 (within the cerebellopontine angle cistern) (3).

**Figure 2:**
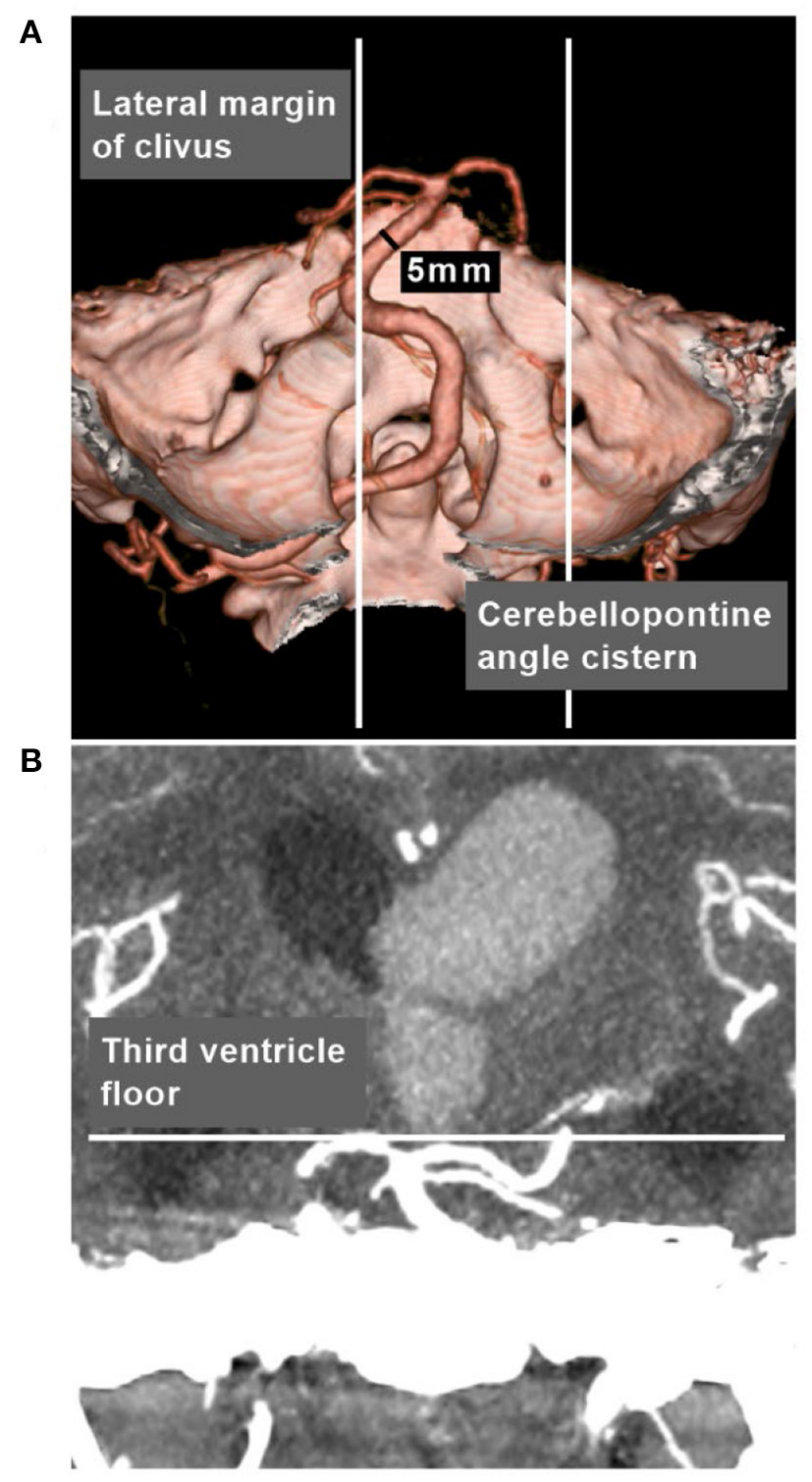
VBD definition in CTA. **(A)** CTA reconstruction image showing the vertebrobasilar arteries were >4.5 mm in caliber at the mid pons level of the BA, and laterally surpassed the lateral margin of the clivus. **(B)** MIP image showing that the BA tip reached the level of the floor of the third ventricle. BA, basilar artery; CTA, computed tomography angiography; MIP, maximum intensity projection; VBD, vertebrobasilar dolichoectasia.

In Smoker's et al. study, the diameter of normal BA averaged 3.17 mm (1.9–4.5 mm), 98% of normal BA courses in the midline or in a paramedian position, medial to the lateral margins of the clivus and dorsum sellae (3). Therefore, it was feasible to define VBD based on the following criteria: (a) the vertebrobasilar arteries were >4.5 mm in caliber at the mid pons level of the BA; (b) the vertebrobasilar arteries laterally reached/surpassed the lateral margin of the clivus or the BA tip reached/surpassed the level of the floor of the third ventricle (Figure 2) (3, 4). That is, VBD should include both the ectasia and lengthening of the vertebrobasilar arteries presented with the lateral, upward or lateral and upward movement of the vertebrobasilar arteries.

### VBD Classification

**Figure 3:**
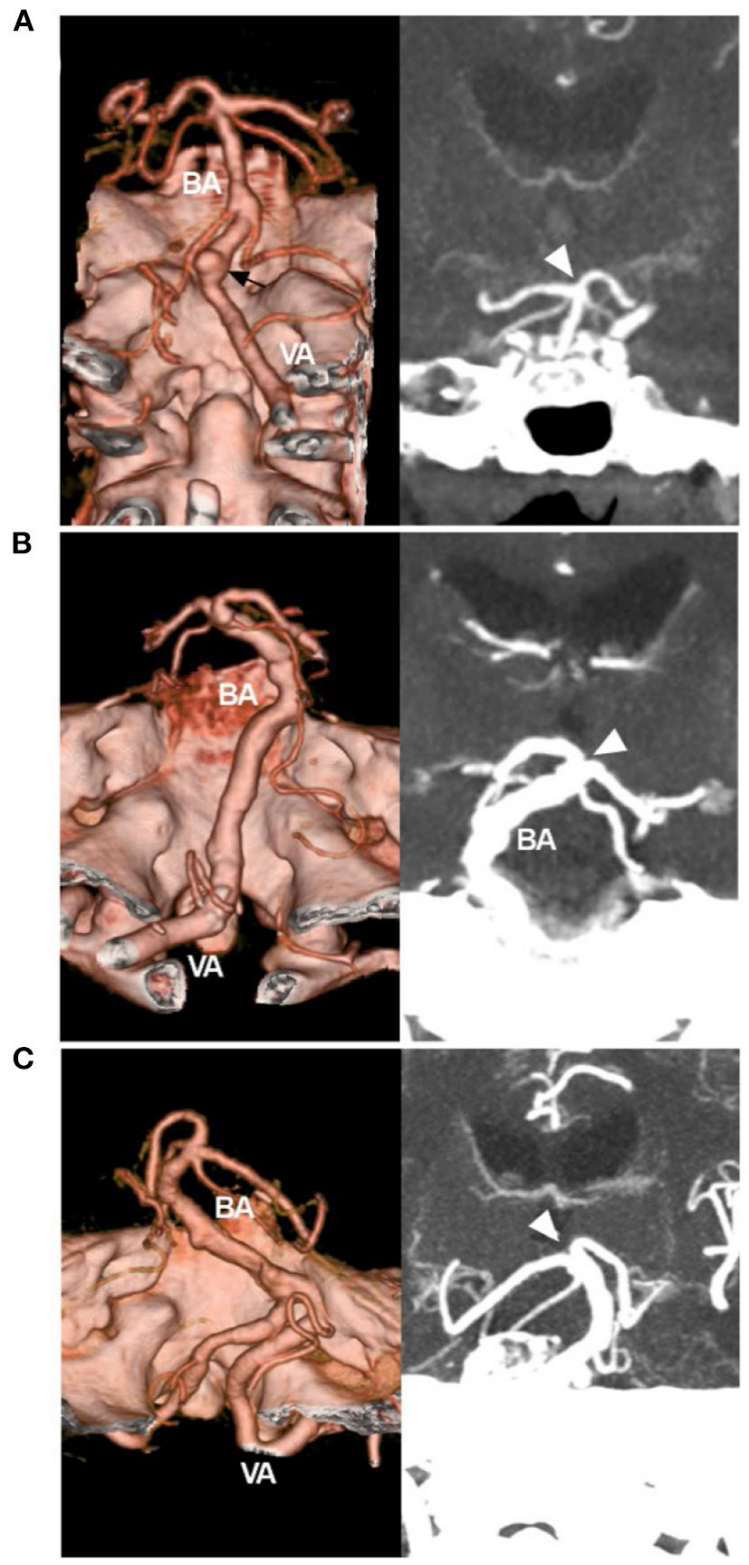
Simplified classification of VBD on CTA. **(A)** Left: CTA reconstruction image showing a mild VBD. The VBD did not reach the lateral margin of the clivus or dorsum sellae, and the arrow indicated an aneurysm. Right: MIP image showing that the BA tip (arrowhead) reached the floor of the third ventricle. The score of the Smoker et al. grading system was 3 (1 + 2). **(B)** Left: CTA reconstruction image showing a moderate VBD; the VBD reached the lateral margin of the clivus or dorsum sellae; right: MIP image showing that the BA tip (arrowhead) elevated the floor of the third ventricle; the score was 5 (2 + 3). **(C)** Left: CTA reconstruction image showing a severe VBD, the VBD reached the cerebellopontine angle cistern; right: MIP image showing the BA tip (arrowhead) elevating the floor of the third ventricle; the score was 6 (3 + 3). BA, basilar artery; CTA, computed tomography angiography; MIP, maximum intensity projection; VA, vertebral artery; VBD, vertebrobasilar dolichoectasia.

CTA can provide more information, including the maximum intensity projection and three-dimensional vessel reconstruction (4, 25). Based on CTA in cases of VBD, according to Smoker et al. the scoring system can be simplified to produce three types of VBD: (1) the mild type has 3 points (1 + 2 or 2 + 1), (Figure 3A); (2) the moderate type has 4–5 points (1 + 3, 2 + 2, 3 + 1, 2 + 3, or 3 + 2) (Figure 3B); and (3) the severe type has 6 points (3 + 3) (Figure 3C).

**Figure 4:**
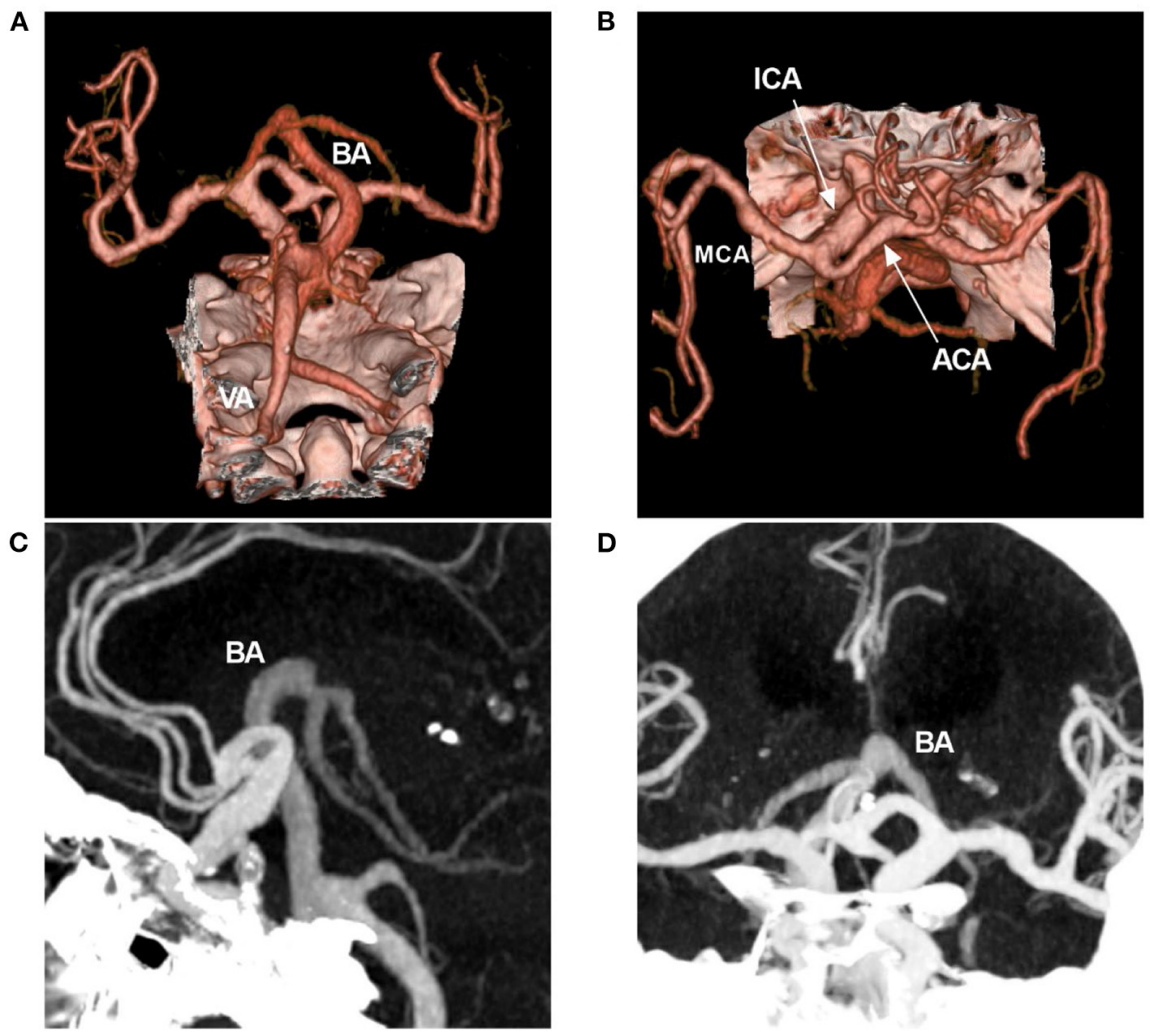
Diffuse intracranial dolichoectasia on CTA. **(A,B)** CTA reconstruction images showing diffuse intracranial dolichoectasia involving the anterior and posterior circulations. **(C,D)** MIP images showing that the BA tip reached the level of the lateral ventricle, the hydrocephalus was found due to the VBD compression. ACA, anterior cerebra artery; BA, basilar artery; CTA, computed tomography angiography; ICA, internal carotid artery; MCA, middle cerebral artery; MIP, maximum intensity projection; VA, vertebral artery; VBD, vertebrobasilar dolichoectasia.

Occasionally, dolichoectasia can affect the anterior circulation, in which diffuse intracranial dolichoectasia is a new, distinct type (Figure 4) (30, 31). Posterior circulation has less sympathetic innervation than anterior circulation, resulting in less trophic support on the vessel wall, which can make the posterior circulation more prone to deformity when exposed to increases in blood flow and pressure (26).

## Natural History

The natural history of VBD varies. It can be stable at a certain stage, presenting with a benign clinical course; it can also progress after a long period of stability and never stop, which is called progressive VBD (32).

In 2008, in the Passero and Rossi study, 156 VBD patients were followed for an average of 11.7 years, and 60% of patients experienced at least one new event, including ischemic and hemorrhagic stroke, compressive symptoms, and hydrocephalus. These events were significantly associated with the severity of VBD. VBD progressed in 43% of patients. The cumulative proportion of survivors who were free of adverse health events was 54.1% at 5 years, 39.5% at 10 years, and 23.5% at 15 years (33).

In 2013, Wolters et al. performed a systematic review involving 375 patients with VBD. The 5-year complications were ischemic stroke in 17.6% of patients, brainstem compression in 10.3% of patients, transient ischemic attack in 10.1% of patients, hemorrhagic stroke in 4.7% of patients, hydrocephalus in 3.3% of patients and subarachnoid hemorrhage in 2.6% of patients; the 5-year mortality was 36.2%, and the prognosis at this time is favorable for patients who are asymptomatic at the time of diagnosis (21).

Other reports of the natural history of VBD had results similar to those of the Passero and Rossi (33) and Wolters et al. (21, 34–37). Did it mean the VBD had a malignant natural history? That is not necessarily the case in the real world. This is because the above previous studies were not randomized trials, and they did not fulfill the criteria to reduce the risk of bias. Therefore, the natural history of VBD needs further investigation (21, 33–37).

**Figure 5:**
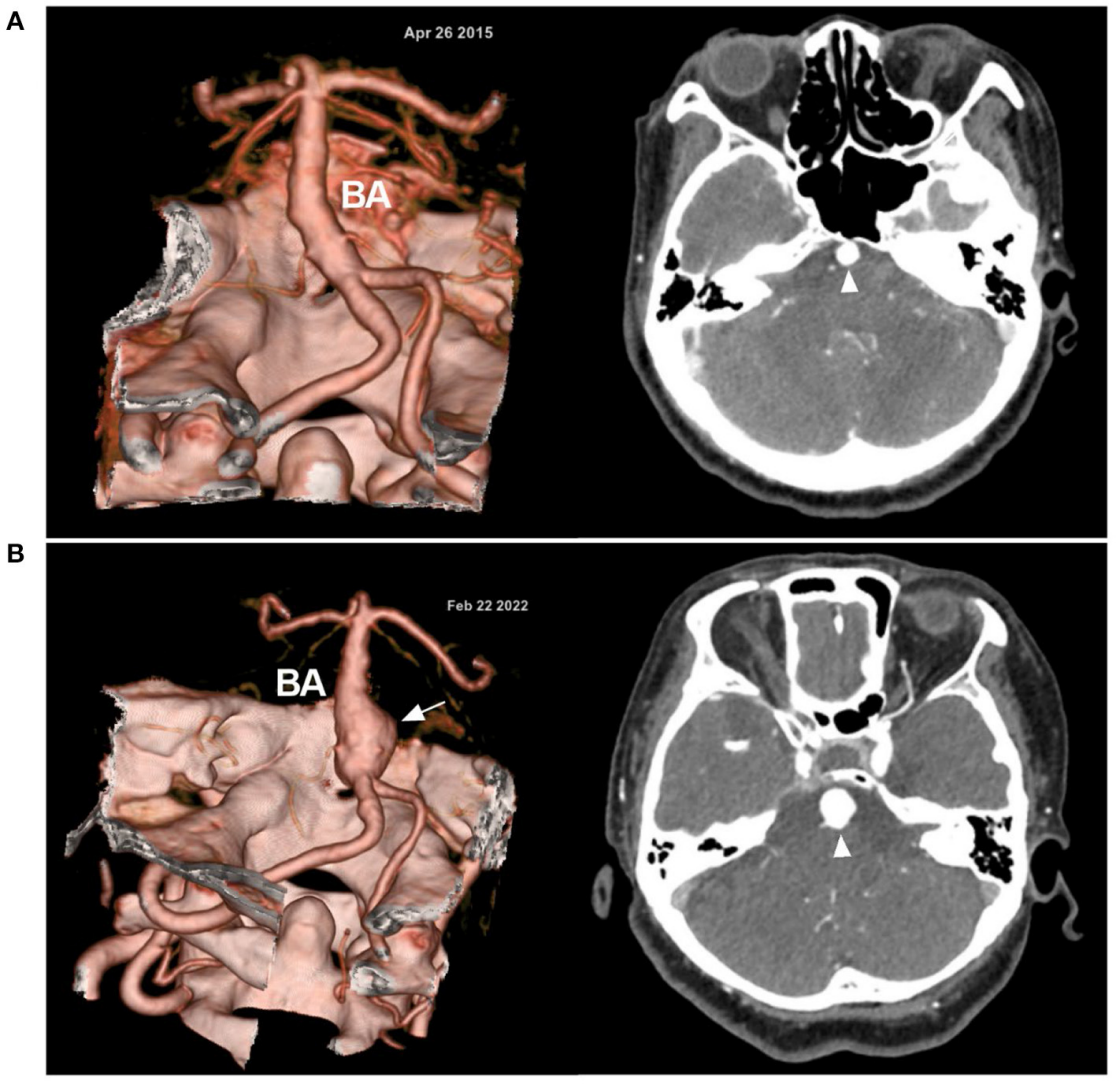
Progressive VBD in CTA. **(A)** In April 2015, CTA (left) showed the VBD; on enhanced CT (right), the BA (arrowhead) is highlighted. **(B)** In February 2022, CTA (left) after 7 years shows the progression of the VBD with the formation of a fusiform aneurysm (arrow); on enhanced CT (right), a larger BA (arrowhead) is highlighted. Over the 7 years, this female patient was always asymptomatic; now she is 61 years old. BA, basilar artery; CT, computed tomography; CTA, computed tomography angiography; VBD, vertebrobasilar dolichoectasia.

**Figure 6:**
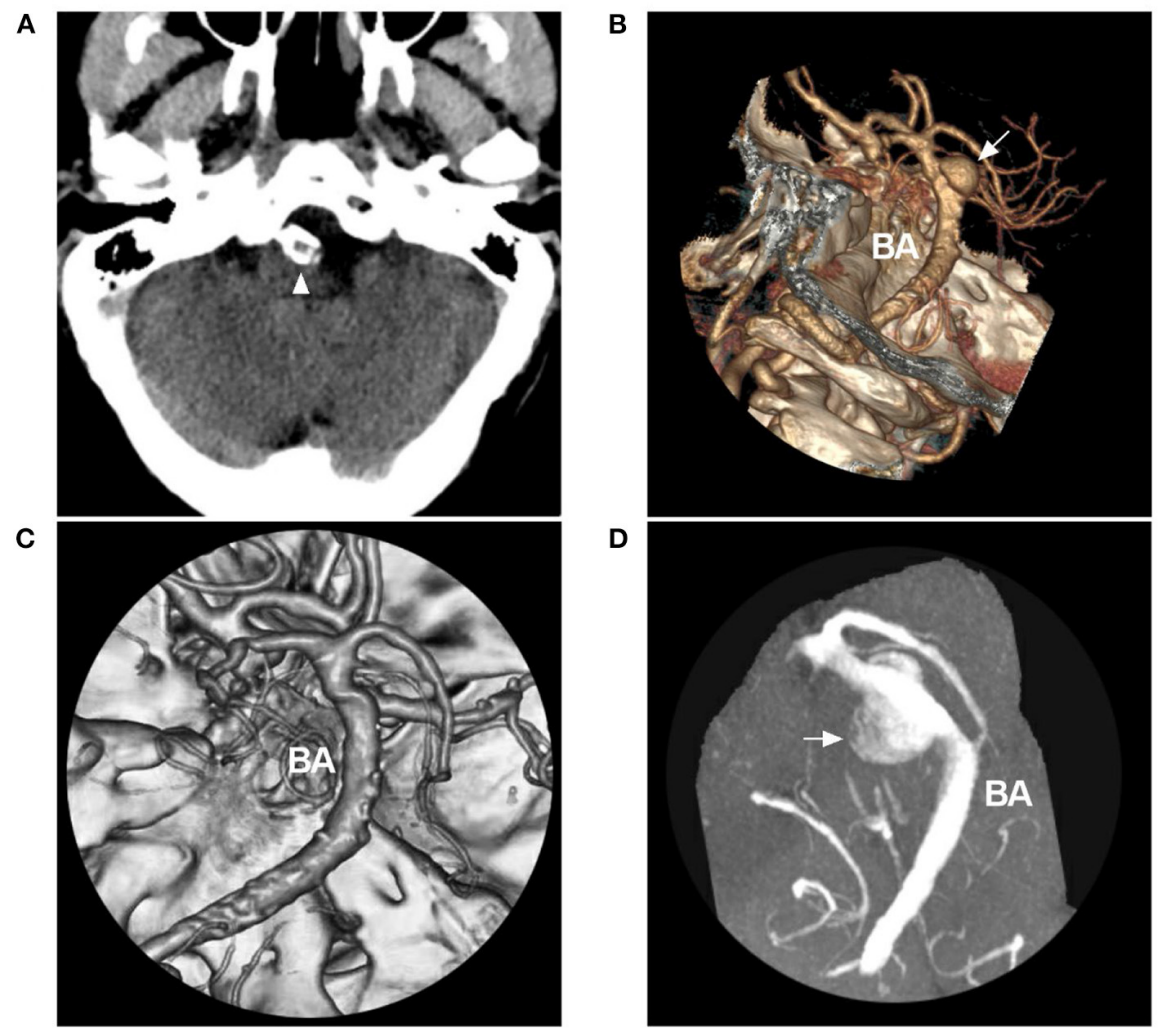
Disguised disappearance of associated aneurysm in VBD. **(A)** CT showing BA calcification (arrowhead). **(B)** CTA showing an aneurysm at the BA (arrow) in a VBD. **(C)** Six-month follow-up CTA suggesting disappearance of the aneurysm. **(D)** Magnetic resonance angiography showing that the disappearance of the aneurysm was disguised due to thrombosis in the aneurysm. The patient suddenly fell into a coma and died within 1 h after 1 month of the last follow-up CTA. Rupture of the aneurysm was considered. BA, basilar artery; CT, computed tomography; CTA, computed tomography angiography; VBD, vertebrobasilar dolichoectasia.

Currently, some consensus is acceptable, that is, that the follow-up complications of VBD are significantly associated with the severity of VBD. Mild VBD has a smooth lumen of the vertebrobasilar artery and can have a benign natural history (28). Some mild VBD can take a period of many years to upgrade to moderate and severe types (5, 13). A progressive VBD after seven years is shown in Figure 5. In moderate and severe cases, VBD patients may be symptomatic and unstable, with stenosis, fusiform dilation or large aneurysms; cases of VBD associated with an aneurysm have a worse natural history (Figure 6) (11, 35, 36, 38).

## EVT Prospects

At present, there is no universally accepted effective treatment for VBD. In the absence of randomized data, EVT is not recommended in most patients with VBD, and among patients with VBD, the high operative risk should be considered against the natural history and clinical context of the presentation.

### Asymptomatic VBD

More than 40% of VBD patients are asymptomatic, although they meet the angiographic diagnostic criteria of VBD (1). Currently, there is no good quality evidence that treating asymptomatic VBD with EVT is better than surgery or better than the natural history of the disease (23, 32). In Wolters's et al. systematic review of 5-year risks in patients who were asymptomatic when VBD was diagnosed, ischemic stroke was 4%, hemorrhagic stroke was 10%, case fatality was 12.5%, and there was no progressive brainstem compression or hydrocephalus (21). Therefore, asymptomatic mild VBD can be left alone; that is, left “untouched.”

When ischemic stroke occurs, antiplatelet treatment can be tried, associating clopidogrel and aspirin for 4 weeks and then long-term aspirin (6). However, antiplatelet or anticoagulant agents are often not as effective as expected in preventing recurrent ischemic events in VBD, which puts them at risk for intracranial hemorrhage (6, 33, 39).

### Symptomatic VBD

Highly selective moderate and severe VBD patients can be EVT candidates, such as those VBD patients with large aneurysms and a greater bleeding risk or those with lower flow conditions and lower velocity, vorticity, and more oscillatory flow (36, 40, 41). EVT for VBD should reconstruct the vertebrobasilar artery to improve blood flow, target the aneurysm to prevent rupture or rebleeding, or reduce the occupying effect. However, overall, it should be managed on a “case by case” basis. Of most importance, the procedure-related morbidity of EVT should be less than the malignant natural history (11).

#### Reconstruction for VBD With Stenting

In theory, stenting reconstruction for VBD could straighten the tortuous vessel to reduce turbulence, restore laminar flow, improve flow in distal branches, provide some degree of flow diversion and reduce the risk of rupture (32, 42). When performing stenting reconstruction, the proximal and distal portions of the stent should cover the normal portion of the artery in the VBD, or telescope stenting reconstruction is necessary (43).

Although, in some reports, the use of stenting reconstruction has shown prospects, the use of stenting reconstruction only appears reasonable as a last resort in VBD patients with a gradually deteriorating condition or cases of VBD complicated by acute dissection (33, 37, 40). In addition, stenting reconstruction should be performed with more caution or should be forbidden in cases of VBD with considerable thrombi in the VBD lumen and for patients with severe or progressive thromboembolic symptoms (41).

Recently, FDSs have become promising for treating VBD. Offering between 24 and 55% metal coverage, the FDS can redirect the blood flow of the VBD (44). The porosity of the FDS is thought to allow blood flow to continue through perforating arteries, given the demand of these vessels (45). FDSs also provide a scaffold for neointimal growth and healing of the vessel wall. Therefore, the use of FDSs may be a curative approach in VBD (23). However, more evidence is needed to confirm this suggestion.

#### Targeting the Aneurysm

**Figure 7:**
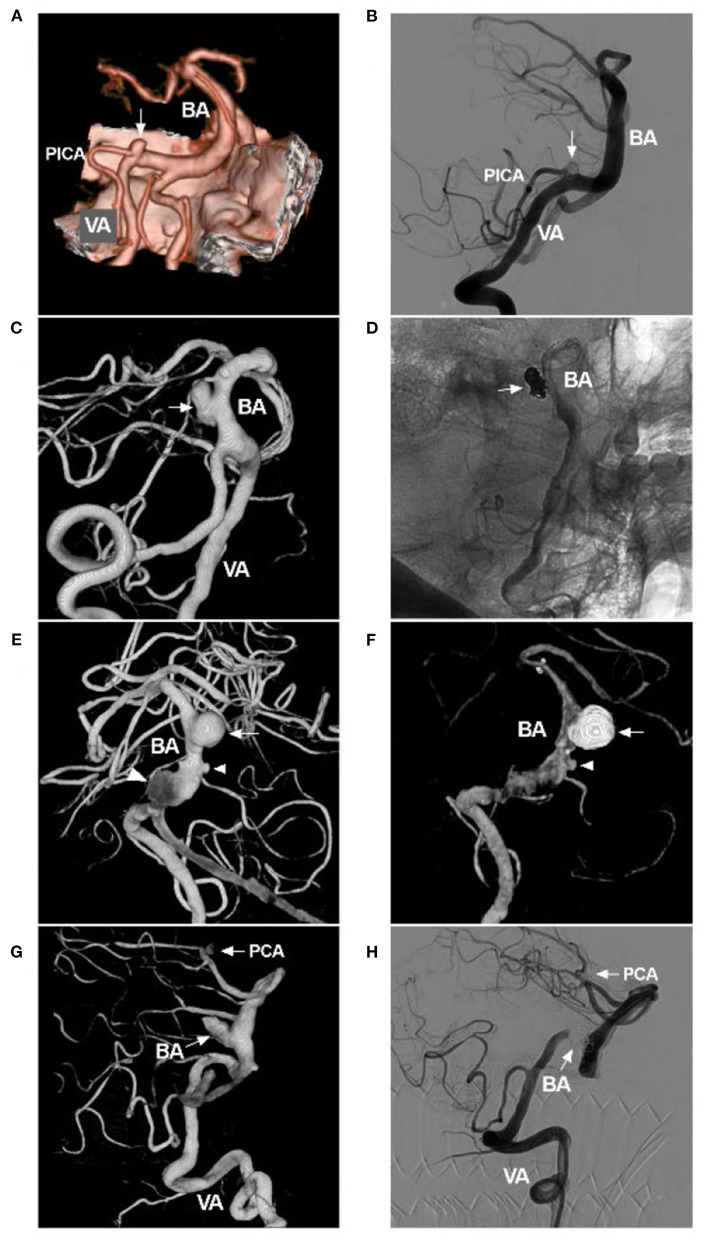
Coiling saccular aneurysms in VBD. **(A)** Pre-operative CTA showing an aneurysm (arrow) of PICA origin in VBD. **(B)** Post-operative DSA showing that the aneurysm (arrow) was coiled with stenting assistance. **(C)** Pre-operative three-dimensional DSA showing an aneurysm (arrow) of the BA in VBD. **(D)** Post-operative DSA showing that the aneurysm (arrow) was coiled with stenting assistance. **(E)** Pre-operative three-dimensional DSA showing multiple tandem aneurysms, a large saccular aneurysm (arrow), a small aneurysm (small arrowhead), and a fusiform aneurysm (large arrowhead). **(F)** Post-operative half-year follow-up DSA showing acceptable embolization with stenting assistance of the large saccular aneurysm (arrow); the small aneurysm did not increase in size. **(G)** Pre-operative three-dimensional DSA showing two aneurysms (arrows with BA and PCA) of the BA and PCA. **(H)** Post-operative DSA showing that the two aneurysms (arrows with BA and PCA) were coiled, the one in the BA requiring stenting assistance. This VBD made treating the PCA aneurysm difficult because of the elongation and tortuosity of the access route. BA, basilar artery; CTA, computed tomography angiography; DSA, digital subtracted angiography; PCA, posterior cerebral artery; PICA, posterior inferior cerebellar artery; VA, vertebral artery; VBD, vertebrobasilar dolichoectasia.

From nature, most aneurysms associated with VBD are dissecting and can present with saccular or fusiform (46, 47). Saccular aneurysms can be treated with the same principle as used for intracranial bifurcation aneurysms (Figure 7). Small fusiform aneurysms in VBD rarely rupture, and they can be given a “watch and wait” approach (41). However, large dolichoectatic aneurysms may be associated with significant morbidity and mortality (35, 41). Steinberg et al. reported that 80% of VBD patients with large aneurysms died or were severely disabled by the 5-year follow-up, especially those demonstrating a mass effect (48). Therefore, EVT would be a meaningful solution for treating these large aneurysms, especially those that have ruptured (29).

Sometimes, the mass effect of the aneurysm of VBD can be improved by inducing aneurysm thrombosis, even without creating significantly noticeable differences in size, suggesting that dampening of pulsatility and vessel wall pressure could be sufficient to alleviate the mass effect (42, 49, 50).

## EVT Techniques

### FDS and Overlapping Traditional Stents

In VBD, FDSs can play two roles. First, FDSs can reconstruct dilated and tortuous vessels; second, they can cure either aneurysmal dilation or associated aneurysms (44, 45). Successful flow diversion should result in progressive aneurysm thrombosis while maintaining side branch patency and requires complete apposition of the implant of the FDS to healthy vessel segments (43).

FDSs will foreshorten significantly because segments affected by VBD are often larger than the largest diameter of the latest FDSs (23). Therefore, large FDSs, such as Surpass (5.3 × 50 mm) (Stryker Neurovascular, Fremont, CA, USA) and Silk (5.5 × 40 mm) (Balt Extrusion, Montmorency, France), are helpful (43). To avoid FDS prolapse, adjunctive coiling of most saccular segments of the VBD can be helpful and act as a scaffold to organize thrombi (51). When the FDS is successfully deployed and steady apposition to the vascular wall is secured, the coil may be left *in situ* or withdrawn to avoid occlusion of the perforating vessels (42).

If one FDS is not sufficient, a second stent can be telescoped into the first FDS. In this situation, efforts should be made to match the overlapping portion of the FDSs with the most severely ectatic segment. The LEO stent (Balt Extrusion, Montmorency, France) had a larger size, such as 5.5 × 75 mm. Therefore, flow diversion using the “diverter-in-stent” technique was helpful. In a report by Cohen et al. after the longest LEO stent was deployed, multiple LEO and Silk stents overlapped (52). After the FDS is deployed, the self-expanding traditional stent can be used to telescope the FDS to cover the proximal and distal vessels, which is a good option to protect the relatively normal vessels (43).

In addition, the use of an FDS may be technically difficult due to the stiffness of the stent and the tortuous path in the VBD. The FDS can be replaced by an LEO stent, with 14% metal coverage, or an LVIS Blue stent (MicroVention, Tustin, California, USA), with 22–28% metal coverage, alone or with coiling assistance (40, 42, 53).

### Coiling Aneurysm With/Without Stenting

In VBD, it is feasible to perform EVT targeting associated aneurysms. Saccular aneurysms with a definite neck can be coiled with/without stenting. EVT for fusiform aneurysms remains challenging (40). For large fusiform aneurysms in VBD, conventional self-expandable stents assisted with coiling have difficulty completely curing these aneurysms, especially those with branches arising from the aneurysmal wall; however, the technique is still useful for target embolization, which targets weak points or blebs ruptured by coiling assisted by conventional stents to await subsequent treatment (54).

**Figure 8:**
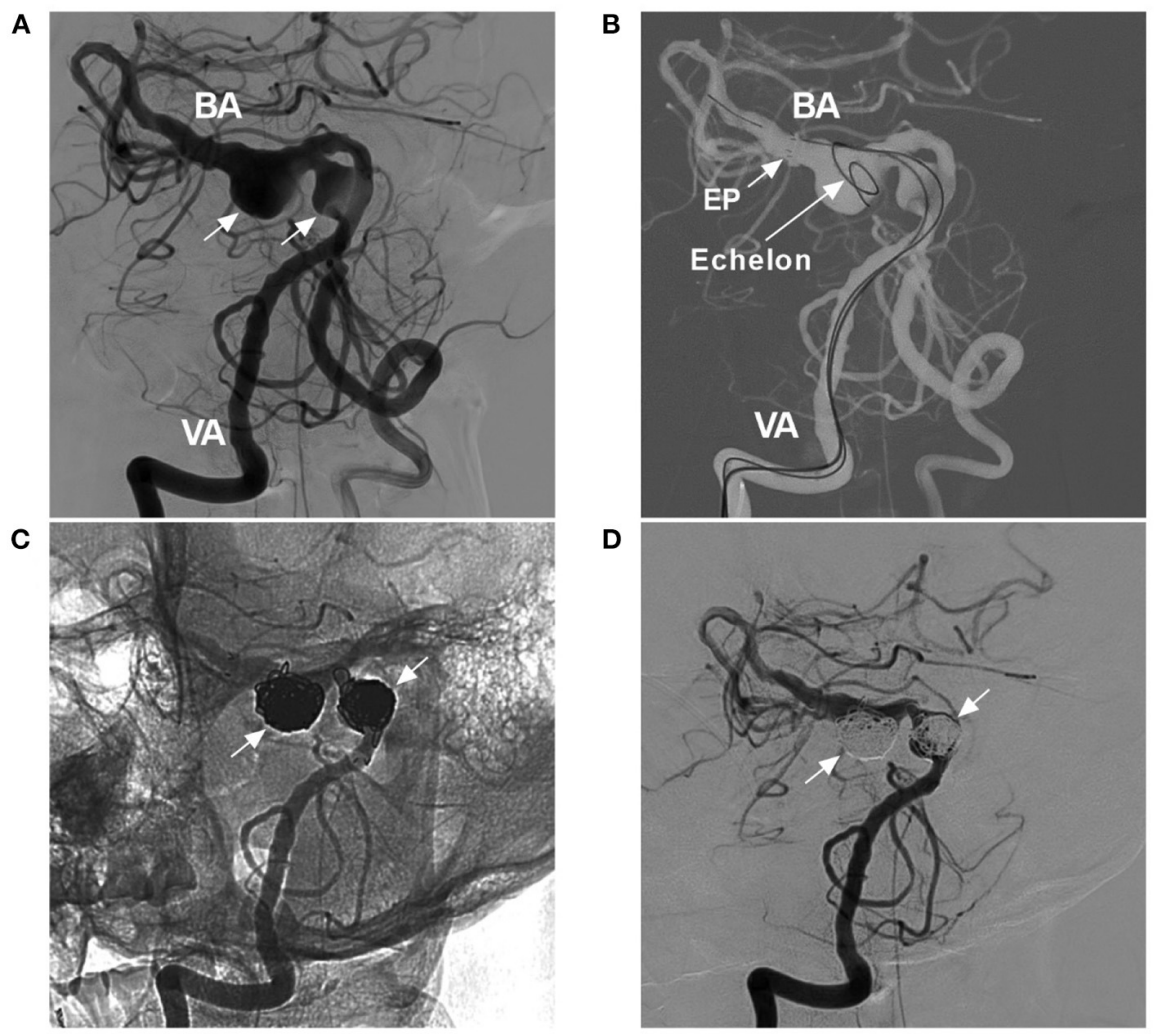
Coiling tandem aneurysms with stent assistance. **(A)** Two-dimensional DSA showing the VBD and two tandem aneurysms (arrows). **(B)** Road map showing the opening of the tip (arrow with EP) of the Enterprise stent (4.5 × 39 mm) and the Echelon-10 microcatheter delivering coils into the aneurysm [arrow with Echelon (Medtronic, Irvine, California, USA)]. **(C,D)** Two-dimensional unsubtracted **(C)** and subtracted **(D)** DSA showing that the two tandem aneurysms (arrows) were coiled under stenting assistance. BA, basilar artery; DSA, digital subtracted angiography; EP, Enterprise stent; VA, vertebral artery; VBD, vertebrobasilar dolichoectasia.

**Figure 9:**
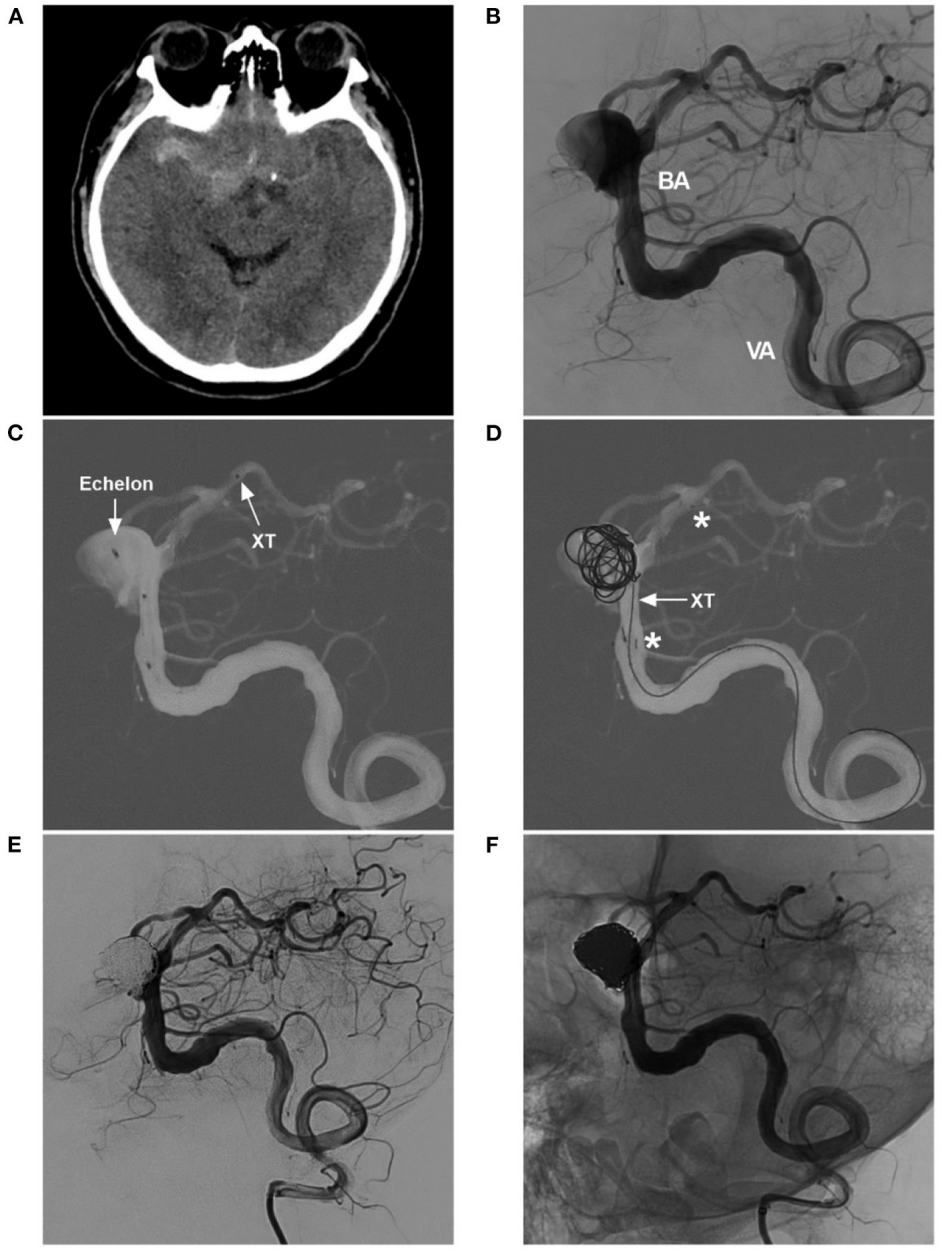
Atlas stent-assisted coiling of an aneurysm in VBD. **(A)** CT showing subarachnoid hemorrhage. **(B)** Two-dimensional DSA showing the VBD and an aneurysm of the BA. **(C)** Road map showing the Echelon-10 microcatheter (Medtronic, Irvine, California, USA) in the aneurysm (arrow with Echelon). The XT-17 microcatheter tip (arrow with XT) (Stryker Neurovascular, Fremont, California, USA) crossed the aneurysm neck and entered the posterior cerebral artery. **(D)** Road map showing an Echelon-10 microcatheter jailed in the aneurysm, which delivered the coils. Then, Atlas stent was deployed; the asterisks indicate the proximal and distal markers. Then, the XT-17 microcatheter (arrow with XT) was guided into the aneurysm by the transcell technique. **(E,F)** Two-dimensional unsubtracted **(C)** and subtracted **(D)** DSA showing that the aneurysm was coiled completely. BA, basilar artery; CT, computed tomography; DSA, digital subtracted angiography; VA, vertebral artery; VBD, vertebrobasilar dolichoectasia.

In stent-assisted coiling, large and long stents can be helpful, especially for tandem aneurysms, such as the largest-size LEO stents (Balt Extrusion, Montmorency, France), Solitaire stents (Medtronic, Irvine, California, USA) and Enterprise stents (Codman Neurovascular, Raynham, MA, USA) (Figure 8). A low-profile Neuroform Atlas stent (Stryker Neurovascular, Fremont, California, USA) can be helpful for passing through highly tortuous vessels (Figure 9).

### Vertebral Artery Occlusion

VBD often involves bilateral VAs. During stenting reconstruction, the non-dominant VA should be occluded immediately after stent deployment to reduce the blood flow into the space between the stents and the vascular wall, which can further improve the hemodynamics of the VBD (55). The occlusion should be made distal to the posterior inferior cerebellar artery, and any branches originating from the VA in these cases must be preserved, especially those of the anterior spinal artery (56).

Currently, parent artery occlusion (PAO) has gradually been replaced by a constructive approach, and PAO is still a useful technique. VBD can be treated by bilateral VA occlusions. In 2003, Omahen and Findlay successfully treated a 48-year-old man with a giant aneurysm in VBD by bilateral microsurgical VA occlusions (57). Therefore, flow reversion and intra-aneurysmal thrombosis after bilateral VA occlusions can be therapeutic options for VBD.

Currently, VA occlusion can be performed safely with EVT, including detachable balloons or coils or with a combination of the two (58, 59). However, two complications after PAO must be considered, thrombotic events and potential hemodynamic insufficiency. Therefore, a balloon occlusion test (BOT) was necessary. Tolerance of bilateral VA occlusion is currently defined as both a lack of new neurological deficits during BOT and angiographic evidence of at least one large posterior communicating artery, as well as evidence of collateral circulation (58). If a patient with VBD could tolerate BOT, bilateral VA occlusion may be performed. To reduce complications, staged occlusion might also be helpful. In addition, both anticoagulant and antiplatelet therapy should be used after the procedure (59).

## EVT Prognosis and Complications

Currently, VBD is still a poorly studied disease. Attempts to stop the progression of VBD by simple stenting reconstruction can often be in vain. For instance, in the O'Shaughnessy report, the VBD still continued to grow despite occlusion of the proximal portions of bilateral VAs; this outcome suggests that the strength of the vascular wall and hemodynamics are both important factors affecting the progression of VBD (60). EVT may be applied to coil ruptured aneurysms or improve blood flow in highly selective cases of VBD.

### Prognosis

Currently, it seems certain that the use of EVT for treating ruptured aneurysms is effective. Only coiling of saccular aneurysms in VBD can result in a good prognosis due to providing the least hemodynamic disturbance. In our previous report, a secular aneurysm on the BA trunk with VBD achieved a good outcome after simple coiling with/without stenting assistance (47). However, for large fusiform and complex aneurysms in VBD, complex EVT, such as FDS deployment, can result in an uncertain and varied prognosis (49).

In a previous small series by Siddiqui et al. 7 large or giant fusiform aneurysms (most of which conformed to VBD) were treated with FDS deployment; 57.1% of the patients had a poor prognosis, and the overall infarct rate was 71.4% (61). In a large series by Bhogal et al. 56 patients with posterior circulation non-saccular aneurysms accepted FDS deployment, in which 58.5% of patients had dolichoectatic and transitional aneurysms. After FDS deployment, in the symptomatic group, 72.7% of patients presented with an mRS score of ≤2, and 54.5% of patients had an mRS score of ≤2 at the last follow-up (23).

Regarding stenting reconstruction for VBD, the reported outcomes of either conventional stents or FDSs vary. He et al. reported that 19 consecutive patients with VBD were treated with 1–3 large LEO stents, and 84.2% of patients had satisfying angiographic reconstruction; the mean mRS score was 3.00 ± 1.15 before treatment and improved to 1.89 ± 1.33 after treatment (42). However, in other reports, the outcomes were disappointing (40, 62). In Wu et al. 9 patients with VBD were implanted with multiple LEO and/or Solitaire stents with or without coil assistance, and only 41.7% of VBD patients demonstrated morphological improvement (40). In Wang's et al. report, 22 symptomatic VBD patients underwent EVT, and only 41% achieved a satisfactory clinical and/or digital subtraction angiography outcome after endovascular treatment (62). Therefore, further studies are needed to investigate this matter.

### Complications

#### Procedure-Related Complications

**Figure 10:**
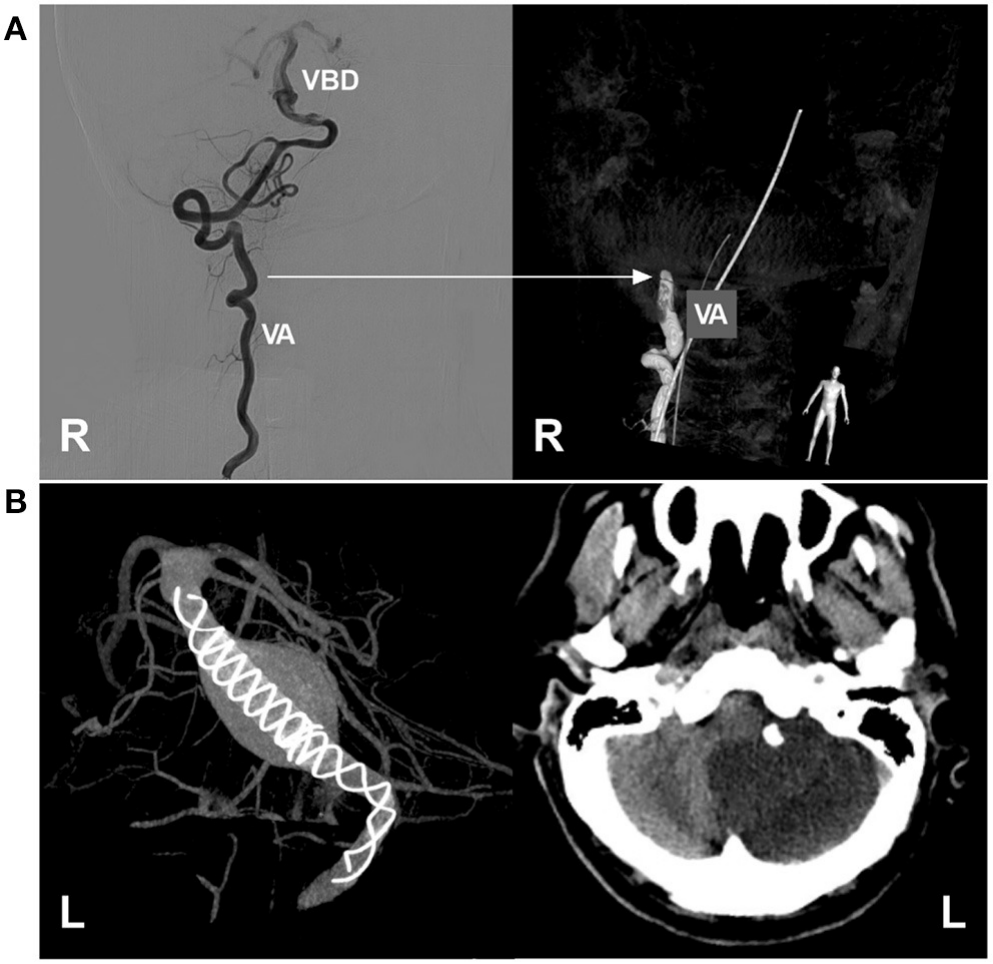
Complications of EVT in VBD. **(A)** Left, two-dimensional DSA showing a VBD with a tortuous right VA; right, three-dimensional DSA showing VA occlusion due to mechanistic dissection during catheterization; the arrow indicates the position of VA occlusion. **(B)** Left, VASO image showing two telescoped TuBridge FDSs (MicroPort, Shanghai, China) implanted in the VBD; right, post-operative CT showing infarction of the left cerebellar hemisphere. BA, basilar artery; DSA, digital subtraction angiography; EVT, endovascular treatment; FDS, flow diverting stent; L, left; R, right; VA, vertebral artery; VASO, vascular space occupancy; VBD, vertebrobasilar dolichoectasia.

EVT in VBD can be accompanied by procedural complications, such as vessel injury during attempted catheterization (Figure 10A), intraoperative aneurysm rupture, and migration of a preexisting thrombus (42). In the report by He et al. the incidence of procedure-related complications was 10.5% (42). Wu et al. reported an incidence of 22.2% (40). Therefore, cautious manipulation in the EVT procedure is necessary.

#### Ischemic Complication

To achieve a sufficient flow diversion effect, in most cases of VBD, two or more FDSs may be needed, and the risk of penetrating branch occlusion will be greatly increased, especially when the adjunctive coils used at the same time cannot be returned, which results in a higher rate of immediate or delayed ischemic complications; penetrating branch infarcts of the brainstem are mostly disabling or fatal (40, 42, 61, 63). In addition to the perforating branch, the large artery can be occluded after FDS deployment (Figure 10B). Therefore, it may be judicious to minimize the number of FDSs deployed and await VBD cure rather than deploy an additional FDS (37). Even if conventional stenting deployment is performed for VBD, such as LEO stents, ischemic complications cannot be avoided completely, and staged stent placement can be recommended (42).

In VBD, the size of the vertebrobasilar artery can often be larger than the diameter of available FDSs, plus an unsmooth lumen of VBD. During FDS deployment, full wall apposition may be difficult, increasing the risks of perforator occlusion or in-stent thrombosis (61). Therefore, “Massage” in FDS with a looping microguidewire tip may be necessary to expand the FDS; if necessary, angioplasty can be used (64).

In VBD, pontine perforators in the fusiform vessel segment of the BA may be occluded and absent, and these pontine perforators must obtain blood flow from the unaffected segment of the BA, as well as from the proximal segments of the anterior inferior cerebellar artery/posterior inferior cerebellar artery and superior cerebellar artery (65, 66). Therefore, during EVT for VBD, these major vessels of the BA and VA should be preserved to avoid brainstem infarction.

During the perioperative period, antiplatelet or anticoagulant agents are necessary to reduce ischemic complications (21, 32, 35). Echiverri et al. considered that anticoagulation might be more effective than an antiplatelet regimen (67). However, more evidence is necessary due to the lack of further controlled studies (35).

Currently, anticoagulant agents are still the mainstream treatment for EVT of VBD. Ruptured VBD can be managed following the EVT principle for intracranial aneurysms, including loading doses of 300 mg/day clopidogrel and 300 mg/day aspirin at least 3 h before the procedure (41). For unruptured VBD, 75 mg/day clopidogrel and 100 mg/day aspirin can be administered for 7 days before the procedure. All patients should be maintained on aspirin and clopidogrel for 1–6 months, followed by indefinite continuation of 100 mg/day aspirin alone (40, 62). Ticagrelor (60 mg twice a day) can be substituted for non-responsive clopidogrel (23).

During EVT, an intravenous bolus dose of heparin (100 IU/kg) was administered, and heparinization was continued to maintain an activated clotting time throughout the procedure two to three times greater than the baseline value (62). After EVT, intravenous heparin with a goal activated partial thromboplastin time of 60–80 s can be continued for 7 days (43).

#### Hemorrhagic Complications

Aneurysms associated with VBD can rupture postoperatively, especially in cases involving FDS deployment (68). In a report by Natarajan et al. a VBD aneurysm ruptured 24–48 h after FDS deployment (63). The rupture after FDS deployment may be explained by a ball-valve-type mechanism with increased stresses in the aneurysm or a substantive inflammatory response that may result in breakdown of the aneurysm wall (68). To avoid delayed rupture of the VBD aneurysm, coils could provide a second layer of protection from hemorrhagic complications, but further evidence is necessary (51).

#### Occupying Effects

For large and giant intracranial aneurysms, after EVT, the aneurysms can enlarge due to thrombosis, and an occupying effect can occur or become aggravated (69). In a report by Wang et al., 22 symptomatic patients with VBD underwent EVT; of them, 13 (54.2%, 13/24) presented with compressive symptoms, and 7 (54%, 7/13) died from severe progressive brainstem compression (62). In addition, even if the lumen of the VBD is reconstructed and improved, the wall of the VBD or aneurysms can continue to expand, resulting in a progressive mass effect (42).

## Summary

For asymptomatic VBD, the “wait and see” or medical management may be a reasonable strategy due to the relatively benign natural history of the disease. EVT may be only effective in highly selected patients. For saccular aneurysms, especially ruptured aneurysms, EVT is reasonable. For fusiform aneurysms, EVT can carry high complication rates and should be recommended with caution. For stenting reconstruction in VBD, the effect was uncertain. For the future of EVT of VBD, a randomized controlled trial is worth pursuing, and might tell us which VBD can benefit from EVT. We also expect the development of neurointerventional products, which will provide more options for VBD.

## Author Contributions

JY contributed to the conception, design of the manuscript, and critically revised the manuscript. JY and YW wrote the manuscript and collected the medical records of the patients. All authors approved the final version of this manuscript.

## Conflict of Interest

The authors declare that the research was conducted in the absence of any commercial or financial relationships that could be construed as a potential conflict of interest.

## Publisher's Note

All claims expressed in this article are solely those of the authors and do not necessarily represent those of their affiliated organizations, or those of the publisher, the editors and the reviewers. Any product that may be evaluated in this article, or claim that may be made by its manufacturer, is not guaranteed or endorsed by the publisher.
